# Protein Antioxidant Response to the Stress and the Relationship between Molecular Structure and Antioxidant Function

**DOI:** 10.1371/journal.pone.0008971

**Published:** 2010-01-29

**Authors:** Rafael Medina-Navarro, Genoveva Durán-Reyes, Margarita Díaz-Flores, Cecilia Vilar-Rojas

**Affiliations:** 1 Department of Experimental Metabolism, Center for Biomedical Research of Michoacán (CIBIMI-IMSS), Morelia, Michoacán, México; 2 Biochemistry Medical Research Unit, National Medical Center, IMSS, México City, México; Griffith University, Australia

## Abstract

**Background:**

Proteins have long been considered a principal target for oxidants as a result of their abundance in biological systems. However, there is increasing evidence about the significant antioxidant activity in proteins such as albumin. It is leading to new concepts that even consider albumin not only as an antioxidant but as the major antioxidant in plasma known to be exposed to continuous oxidative stress. Evidence presented here establishes a previously unrecognized relationship between proteins' antioxidant capacity and structural stress.

**Methodology/Principal Findings:**

A chemiluminiscence based antioxidant assay was achieved to quantify the antioxidant capacity of albumin and other proteins. The capabilities of proteins as antioxidants were presented, but in addition a new and powerful component of the protein antioxidant capacity was discovered. The intrinsic component, designated as Response Surplus (RS), represents a silent reserve of antioxidant power that awakens when proteins face a structural perturbation (stressor) such as temperature, short wave UV light, the same reactive oxygen species, and more extreme changes like glucose or aldehyde-mediated structural modifications. The work also highlights the importance of structural changes in protein antioxidant properties and the participation of sulfhydryl groups (SHs) in the RS antioxidant component. Based on recent evidence about the SH group chemistry, a possible model for explaining RS is proposed.

**Conclusions/Significance:**

The data presented show the significant antioxidant behavior of proteins and demonstrate the existence of a previously unrecognized antioxidant response to the stress. Several implications, including changes in elementary concepts about antioxidants and protein function, should emerge from here.

## Introduction

A now old definition attempts to define an antioxidant as “any substance that, when presented at low concentration compared with those of an oxidized substrate, significantly delays or prevents oxidation of the substrate” [1]. Over the years this definition has come to be recognized as “clearly imperfect” [2] because it excluded proteins such as albumin and others to be considered strictly as antioxidants. A new, much more general concept defined antioxidant as “any substance that delays, prevents or removes oxidative damage to a target molecule” [3]. Albumin thus has been considered not only as an antioxidant, but as the major circulating antioxidant in plasma known to be exposed to continuous oxidative stress [4], [5]. From the perspective of a new concept, it has been possible to find extraordinary characteristics among the antioxidant properties of the proteins.

Several studies have shown direct or indirect reactive oxygen and reactive nitrogen species (ROS/RNS) scavenging properties of human and bovine albumin. Some of these properties, many of which rely on molecular structure, include: 1. The binding of bilirubin at Lys 240 position [6], which was shown to act as a co-antioxidant with α-tocopherol to inhibit lipid peroxidation [7]; 2. The copper and iron variety of binding sites of the albumin molecule, which results in an antioxidant capacity observed only in preventive or primary antioxidants such as ceruloplasmin and ferritin [8], [9]; 3. The SH group of albumin derived from cysteine 34 that represents an important antioxidant reserve to scavenge hydroxyl radical and peroxinitrite [8],[10]; 4. The oxidation of methionine residues in albumin could constitute a scavenging system that serves as an antioxidant reserve which in addition can be recycled [11], although more recent studies have concluded that methionine works mainly as a metal chelator [12].

The combination of these particular properties may result in the general contribution of proteins to the total antioxidant capacity of human blood plasma. This contribution could account for more than 50% of the combined antioxidant effects of urate, ascorbate and vitamin E in blood plasma [13], [14]. Albumin in particular exhibits antioxidant capabilities, and some of them, which are closely linked to its molecular structure, may also be present in many other proteins.

In the past, some studies have proven the loss of antioxidant activity of albumin as a consequence of structural changes [15], [16], [17]. However, a positive change in antioxidant activity as a functional consequence of structural changes of a protein has not been considered until now. Furthermore, the changes adduced by previous reports were clearly linked to the passive red-ox state of the thiol groups and particularly to the albumin red-ox active thiol group (Cys-34).

The present work shows evidence of an intrinsic property of proteins that we have named Response Surplus (RS). RS enables the albumin in particular, but possibly all proteins in a broader context, to overcome the characteristics of the conventional antioxidants, responding to a structural stressor with an increase in their antioxidant potential. The present work emphasizes the close relationship that exists between the antioxidant capacity of proteins and their molecular structure. In addition, we present a possible explanation of the way in which proteins undergo the RS, supported by recent evidence relative to the sulfenic acid formation and the hypothetical intervention of a driven force moving the antioxidant capacity of proteins toward their more energetically favorable native protein configuration, in accordance with Anfinsen's thermodynamic hypothesis [18], [19].

## Results and Discussion

### Protein Antioxidant Capacity (AC) and Protein Response Surplus (RS) Definitions

In the present work, for the first time the expression protein Response Surplus or RS is introduced for the purpose of designating the increase of antioxidant capacity of a protein when it undergoes the effect of a structural stressor. With the objective of obtaining a measurement of the RS, the antioxidant capacity (AC) of a protein sample was determined before (ACb) and after (ACa) an oxidative or structural challenge; AC and ACb are then equivalents. The results obtained in Trolox concentration (Trolox Equivalent Units, nM TEU) with the use of standard curves are transformed to %, considering ACb to be 100%. In this way the values reported as RS represent the percentage of antioxidant capacity surplus above the average antioxidant capacity of proteins before treatment (native protein). In the same way the antioxidant capacity accumulate % (ACA %) represents the sum of antioxidant capacity AC plus the RS value in percentage. In the present work, the ACA % appears above the bars as % or as a series of % values, considering the average antioxidant capacity of the native sample tested to be 100%. In this manner, a quick way to obtain RS from the results is to subtract 100 from ACA %.

AC, Antioxidant Capacity. Trolox nM concentration (Trolox Equivalents Units, TEU)

ACA%, Antioxidant Capacity Accumulate %  =  (AC after) (100) ÷ AC before

RS%, Response Surplus %  =  (ACA %)−100

### Structural Stressors on the Antioxidant Capacity (AC) and Response Surplus (RS) of Albumin

**Figure 1:**
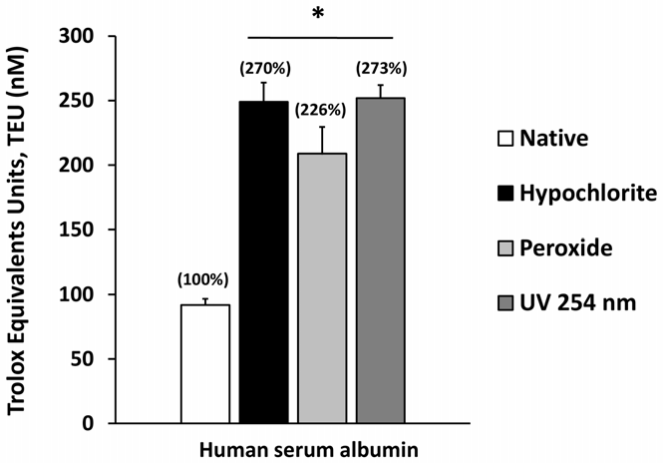
The Antioxidant Capacity (AC) of albumin and the Response Surplus (RS) as a novel component of the albumin antioxidant properties induced by structural stress. The antioxidant capacity was measured before and after albumin incubation with sodium hypochlorite (NaOCl) and hydrogen peroxide (H_2_O_2_) for 10 minutes and exposure to UV light for 15 seconds. The Antioxidant Capacity Accumulated (ACA %) appears above the bars. RS values correspond to ACA%-100 as specified in the Material and Methods section. Results are expressed as mean ± SD (n = 6). *P<0.001 vs. Native albumin.

Antioxidant capacities (AC) were measured and Response Surplus (RS) calculated in human serum albumin (HSA) with the use of a chemiluminiscence system described in the Material and Methods section, and the results are summarized in Figure 1. The native HSA showed an average AC of close to 90 nM TEU (Trolox Equivalent Units), but it more than doubled when the protein was previously incubated for 10 minutes with two strong oxidants: sodium Hypochlorite (NaOCl) 2 mM and hydrogen peroxide (H_2_O_2_) 500 µM (250 and 225 nM TEU respectively). The same pattern was obtained when protein was exposed to UV light (254 nm) for 15 seconds (p<0.01) (250 nM TEU). The Antioxidant Capacity Accumulated in % (ACA %) corresponds to 270%, 226%, and 273% respectively, and appear in parentheses above the bars. RS % corresponds to ACA % -100, as was described previously. There are significant differences between the treated groups and the native protein antioxidant capacity (p<0.001), but there are no significant differences between the treated groups in terms of TEU, ACA % or RS % values.

**Figure 2:**
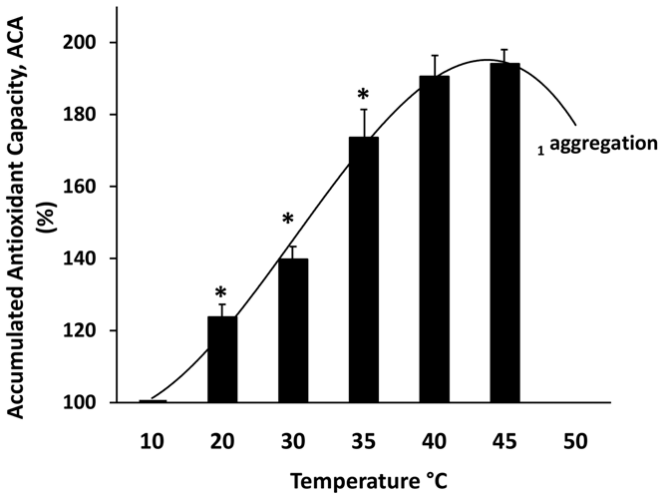
The antioxidant behavior of Human Serum Albumin (HSA) in a pre-denaturation temperature thermal gradient. The Accumulated Antioxidant Capacity (ACA%) appears as a function of the thermal change. Each point corresponds to an independent experiment verified at indicated temperatures. The antioxidant capacity (AC) of the albumin placed at 10°C was 48 nM TEU and corresponds to 100% of ACA%. From this point the antioxidant capacity of albumin increases with the temperature, until it reaches almost 200% ACA. RS correspond to ACA%-100 as specified in the Material and Methods section. Above 45°C it is possible to observe protein aggregation and light scattering at 400 nm in the stock solutions. Each point represents the average of three separate experiments ± SD. All points with a P<0.001 vs. 10°C temperature (ACA = 100%). *P<0.001 vs. previous and next point. ^1^Temperature for aggregation is concentration-dependent (more details in the text).

Such as oxidative stress, thermal treatment produces changes in the AC and RS of albumin. The thermal denaturation process of HSA is comprised of two stages [20]. The first is a reversible thermal structural alteration of the protein, and the second a state that results in an irreversible structural change at different temperatures dependent of albumin concentration [21]. We find albumin aggregation (light scattering at 400 nm) close to 50°C with an albumin concentration of 100 mg/ml. In the present work, with the purpose of testing the antioxidant properties derived from thermal structural changes, we studied the antioxidant behavior of albumin in a temperature range of 10 to 50°C. The results demonstrate clearly that albumin changes its antioxidant properties within a reversible thermal denaturation process in a temperature-dependent manner (Figure 2). When the native HSA was set at 10°C, it yielded an AC of close to 50 TEU (which corresponds to 100% of ACA in the graph). From this point the antioxidant capacity increased gradually with the temperature, until it reached almost 200% ACA as it approached 50°C, just before it was possible to see evidence of aggregation. From this point the antioxidant capacity apparently diminished, probably as a consequence of the formation of intermolecular disulphide bridges by free SH groups, which plays an essential role in aggregation process [21]. However, some extent of chemiluminiscence light interference with turbidity cannot be discarded.

**Figure 3:**
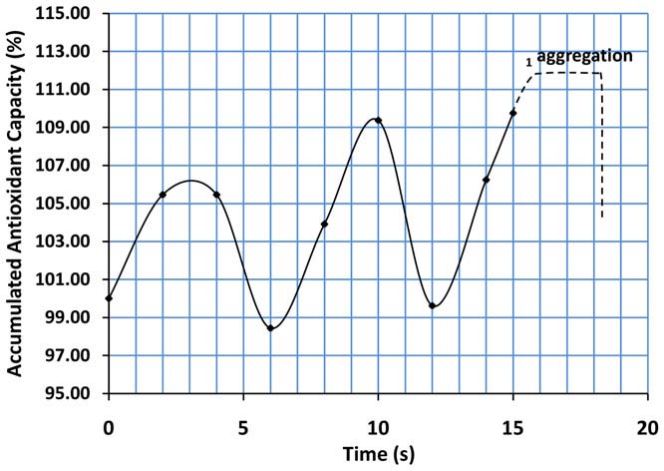
Antioxidant changes (RS) induced previous thermal denaturation. A cyclic pattern of antioxidant changes may be observed just before protein aggregation. Protein stock solutions (100 mg/ml) were placed at 65±0.1°C and samples were collected several seconds before the denoted protein aggregation (scattering at 400 nm). A characteristic cyclic pattern of increased and decreased antioxidant capacity could be observed before protein aggregation. The antioxidant capacity (AC) of the albumin previously placed at 65°C was 94 nM TEU and corresponds to 100% of ACA%. RS correspond to ACA%-100 as specified in the Material and Methods section. The increase and decrease of ACA did not exceed 10% of the base antioxidant level. The graph corresponds to a representative experiment.^ 1^Temperature for aggregation is concentration-dependent (more details in the text).

In independent experiments, when the denaturation temperature was maintained at 65°C, the antioxidant capacity showed a particular and repetitive pattern of fluctuation several seconds before protein aggregation and light scattering at 400 nm (Figure 3). We speculate that the fluctuations observed could correlate with a transient fluctuation pattern of structural unfolding and refolding. However, in contrast to the ACA of close to 200% at about 50°C (Figure 2), the changes observed here never exceed 10% of ACA.

### Importance of Structural Integrity on the Antioxidant Capacity of Albumin

**Figure 4:**
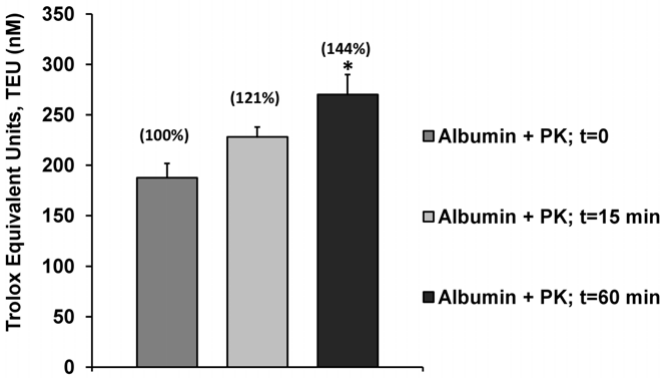
The effect of hydrolysis of albumin on Antioxidant Capacity (AC) and Response Surplus (RS). The antioxidant capacity of albumin was increased with the hydrolysis procedure with Proteinase K (PK). However, the magnitude of the antioxidant capacity before 15 minutes and 60 minutes (total hydrolysis) of incubation with PK were only 1.2 and 1.4 times the combined original antioxidant capacity of albumin and PK without incubation (t = 0). The Antioxidant Capacity Accumulated (ACA %) appears above the bars and corresponds to 0, 15 and 60 minutes of incubation time respectively. RS values correspond to ACA % - 100 as specified in the Material and Methods section. Results are expressed as mean ± SD (n = 5). *P<0.05 vs. native albumin.

Experiments to explore the effect of proteolysis and hence the structural integrity of the AC and RS of albumin showed that AC is increased as a result of hydrolytic activity of proteinase K (Figure 4). However, it was clear that the magnitude of the response was partial compared to the result observed for normal albumin (see Figure 1). Incubation of 15 minutes with proteinase K increases the AC from 187 (native) to 228 (treated) TEU (21% of RS) and 60 minutes of incubation with the proteinase K changes the AC from 187 (native) to 270 (treated) TEU (44% of RS). This means that responses of 1.21 and 1.44 times those of native albumin (including the contribution of the same proteinase K) were obtained. Although 60 minutes of incubation with proteinase K virtually totally hydrolyzed the albumin in the experimental conditions used, the maximum antioxidant capacity achieved in this way does not explain the magnitude of the change in AC reached with stressors like Reactive Oxygen Species (ROS) and UV light (Figure 1). It is possible to suppose that with the hydrolysis reactive residues from the structure of a protein such as the SHs groups could be liberated, promoting reactions of these with free radicals as described in the next section. In any case, these results implied that structural molecular integrity is important to proteins if they work as antioxidants (AC) and when they face a stressor (RS).

### Contribution of the SHs Groups to the Protein Antioxidant Capacity (AC) and the Response Surplus (RS) Component

**Figure 5:**
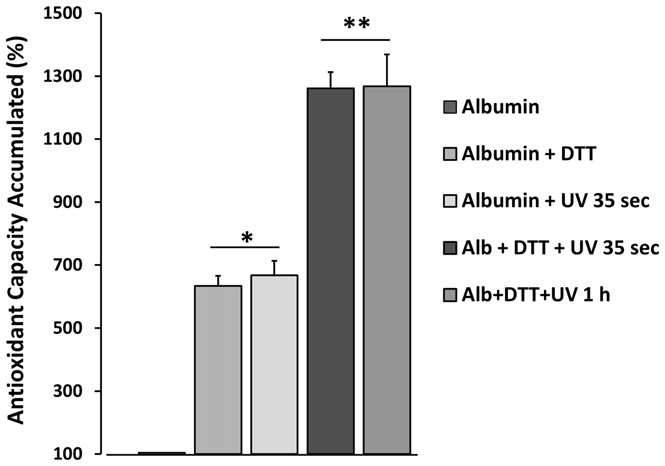
Contribution of thiol groups to the albumin antioxidant Response Surplus (RS) component. Reduced albumin and albumin exposed 35 seconds to UV light (254 nm) presented equivalent changes in antioxidant capacity (ACA% around 600%). However, when samples reduced previously with DTT were exposed to UV light for 35 seconds, it was possible to observe an additive effect (ACA% around 1300%). Additional exposure time to UV light does not produce additional antioxidant changes. Results are expressed as mean ± SD (n = 5). * p<0.005 vs. native albumin; ** p<0.001 vs. albumin + DTT and albumin + UV light 35 sec.

It has been proposed that in living cells the protein thiols could be directly involved in the cellular defense mechanism against oxidants [22]. However, that would imply that sufficient SHs were available to be oxidized. New reports confirm that most cellular protein SHs equivalents are found in the reduced state and that protein thiols are directly involved in the cellular defense mechanism against oxidants [23]. In the present study, the contribution of the reducing equivalents on the antioxidant capacity of albumin was achieved by using the reducer agent DTT (Figure 5). As was expected, results indicated that reduced albumin increased its AC to a high magnitude (ACA% = 600%); an equivalent amount was reproduced with an exposure of 35 seconds to UV light. It is noteworthy that the partial contribution of both stressors and the cumulative character of these effects (DTT+UV) reached an antioxidant capacity close to 13 times that of normal albumin. This data indicate that although SHs groups are important reactive residues and key to the RS phenomenon, the stressor (and the structural response induced on proteins) is necessary to explain the origin of the antioxidant capacity remaining after DTT exposure. An hour of UV light (plus DTT) did not further increase the antioxidant capacity.

**Figure 6:**
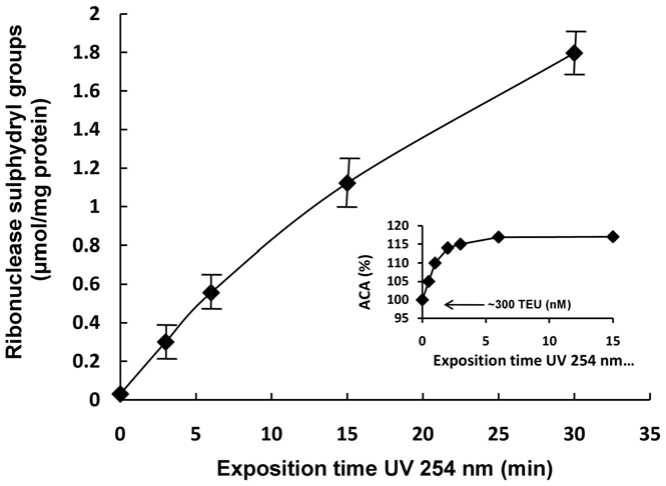
Thiol groups in Ribonuclease A exposed to UV light and antioxidant behavior of the protein. UV light (254 nm) exposed thiols from disulphide's ribonuclease groups in a time dependent manner. In the internal graph, ribonuclease in the native state showed an elevated antioxidant capacity (∼300 TEU, nM), but in contrast, the change in antioxidant capacity represented as ACA% after UV light exposure was relatively short and did not exceed 116–120% independent of the exposure time above 5 min. Results are expressed as mean + SD of three experiments. The internal graph corresponds to a representative experiment.

With respect to the previous results, in the present work RNase A (ribonuclease A, 124 residues, ∼13.7 kDa) was used as a means of understanding better the nature of RS and its relation with the SHs groups in the presence of UV light as stressor (Figure 6). The antioxidant capacity of ribonuclease challenged with UV light yielded relatively low RS %, reaching around 16% with 5 minutes of exposure time (ACA% = 116%), but surprisingly, ribonuclease in native state presented a high level of AC (300 nM TEU) (Figure 6, internal graph). It is noticeable because in this state ribonuclease does not normally present free SHs groups. Indeed, when an attempt was made to measure the SHs, detectable amounts were not obtained (Figure 6, t = 0). These results indicated that the stressor liberates SHs groups from the protein structure.

The behaviors of ribonuclease in relation to a high AC and relatively low RS can be explained if one aspect of the system is taken in account: all systems for measuring the antioxidant activity are sources of free radicals and stressors. It is possible to hypothesize that some proteins should be more sensitive to the free radicals derived from the same system. If the former is true, a rapid induction of RS could be produced when the ribonuclease is placed with the reaction mix. This would be an unavoidable consequence of the use of the system for some proteins. However, it seems clear that passive intervention of SHs groups does not by itself explain the high antioxidant capacity observed (around 300 TEU) because there were no detectable free SHs groups in native ribonuclease.

### AC and RS of Three Non-Related Proteins

**Figure 7:**
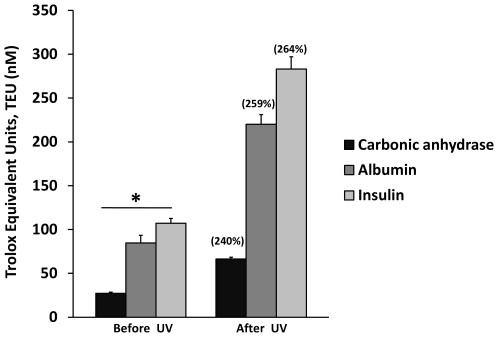
Antioxidant Capacity (AC) and Response Surplus (RS) of three not closely related proteins. Carbonic Anhydrase (CA), Bovine Serum Albumin (BSA) and Insulin (I) presented different AC and relatively similar RS after being exposed to UV light as structural stressor. The AC of the proteins was measured before and after exposure to UV light for 1.5 minutes. Antioxidant capacity changed on average from 27, 84, and 107 nM TEU to 66, 220 and 283 nM TEU (CA, BSA and I respectively). The percentages in parentheses above the bars correspond to each ACA% value. Results are expressed as mean ±SD (n = 5). P<0.01 when compared before and after UV light exposure.

With the objective of looking for quantitative differences from other proteins in relation to AC and RS, three non-related proteins were tested and the results are presented in Figure 7. The AC resulted in 27 nM TEU for the carbonic anhydrase (Isoform CA-I, 29 KD), 85 nM TEU for the Bovine Serum Albumin (Fraction V, 66.4 KD) and 106 nM TEU for Human Insulin (recombinant 5.8 KD). The change of AC by exposure to UV light (254 nm) for 1.5 minutes resulted in 66, 220 and 283 TEU for Carbonic anhydrase, BSA and Insulin respectively. The corresponding values of ACA % for the three proteins in the same order were 240%, 259% and 264% (shown above the bars in Figure 7), and correspond to % of RS of 40, 25.9 and 26.4. There are significant differences between the antioxidant capacities of the proteins before and after exposure to UV light (p<0.01). No correlation between the antioxidant capacity and the molecular size or amino acid sequence of the proteins tested could be identified. However, it is possible to see first, an inverse relationship between antioxidant capacity and the protein's half life (Figure S1) and second, a correlation between antioxidant capacity and the total amount of cysteine residues per gram of protein calculated (Table S1). However, the former result does not explain completely the differences presented in antioxidant capacity, because no single or minimal amount of cysteins is normally presented in reduced state. As in the previously explained case of ribonuclease, we do not exclude the possible intervention of the ROS derived from the system in the induction of the antioxidant response. We hope data presented here will stimulate interest in investigating this aspect more deeply.

### Chemical Modifiers of Albumin Increase AC but Reduce the RS Even More

In the present work two chemical modifications of albumin were tested to learn how the changes introduced affect the antioxidant capacity and RS in response to a stressor such as UV light. Incubation of albumin with acrolein results in the adduct formation with lysine [24], whereas incubation of albumin with glucose produces several modifications to protein residues grouped under the term glycation or non-enzymatic glycosylation. Previous reports of in vitro modifications of bovine serum albumin (BSA) by methylglyoxal [15] and glucose [16] indicate that these modifiers decrease content of the sulphydryl groups, impairing the antioxidant properties of albumin and inducing conformational changes attributed to diminishing of the fluorescence of the tryptophan residues.

**Figure 8:**
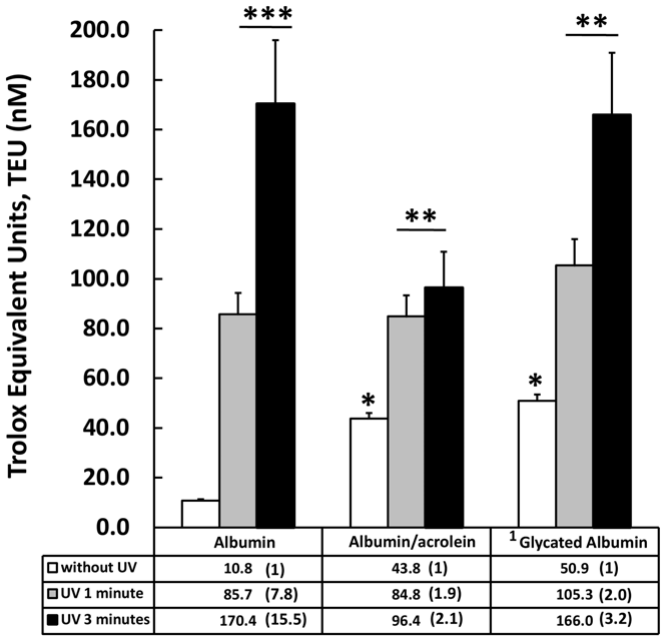
Structural modifications produced by acrolein and glucose on the Antioxidant Capacity (AC) of albumin. Acrolein and glucose partially glycated produce an increase in the AC of albumin of up to 4 times the control native albumin (white bars) value. The exposure of UV (254 nm, 1 and 3 minutes) induces significant increases of AC of albumin exposed with respect to the control. However, the change produced in native albumin was higher than that presented by modified ones. The table below the graph includes the average AC of control samples and treated with UV (values in table without SD), and in parentheses the number of times the antioxidant capacity (AC) of albumin samples were increased in relation to the respective control. Results are expressed as mean ± SD (n = 5). *P<0.005 vs. control native albumin without UV; **P<0.005 vs. control (without UV); ***p<0.001 vs. control (without UV). 1 Partially glycated as described in the Material and Methods section.

**Figure 9:**
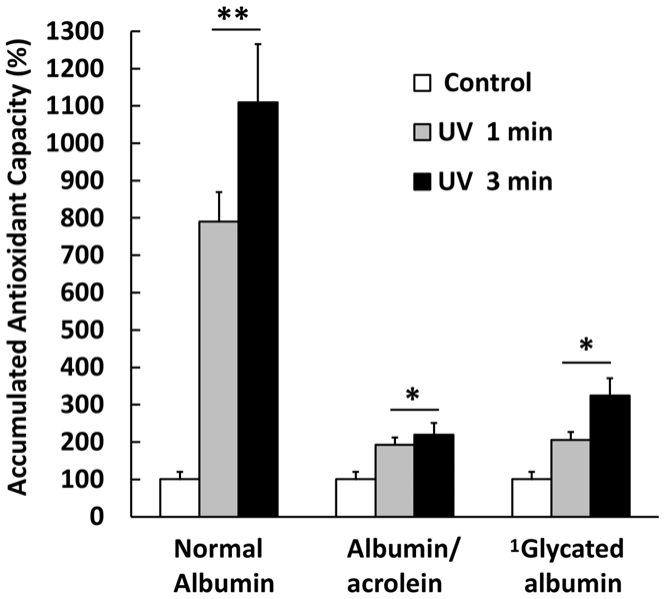
Response Surplus (RS) produced by albumin modified by acrolein and glucose. Structural modifications introduced by acrolein and glucose impaired the RS of the protein by a high magnitude. Normal albumin exposed to UV light responds to the stressor with an increase of 8 and up to 11 times the base value (100%) even before 1 and 3 minutes of exposure respectively (ACA% = 800% and 1100%). In contrast, samples of albumin modified with acrolein and partially glycated respond with increases of 2.2 and 3.2 times the base value of 100%. Base value corresponds to the Antioxidant Capacity average before exposure to UV light, and appears as 100% in the graph. Results are expressed as mean ± SD (n = 5). *P<0.005 vs. albumin control; **P<0.001 vs. native albumin. 1 Partially glycated as described in the Material and Methods section.

The evidence indicates that the protein modifiers used here increase the antioxidant capacity of the albumin (Figure 8). The structural changes produced by protein-acrolein adduct formation and the non-enzymatic attachment of glucose to free primary amine residues seem to be stressors capable of inducing an incipient antioxidant response, increasing the AC by up to 4 times the native albumin. In contrast, these same changes produced a drastic reduction in the RS of the albumin (Figure 9) when it was previously challenged with the exposure of UV light (254 nm). One minute of exposure to UV light increased the ACA % 8 times, to 800%, whereas albumin treated previously with acrolein and glycosilated albumin in the same conditions increased the ACA % by only 1.9 and 2 times respectively (ACA% = 190 and 200%). When the previous exposure time to UV light was increased to 3 minutes native albumin reached more than 11 times the antioxidant capacity of native albumin (ACA% = 1100), whereas acrolein-modified and glycosilated albumin achieved only 2.2 and 3.2 times the antioxidant capacity presented before UV exposure (ACA% = 220 and 320% respectively). The results previously described emphasized the importance and complexities of the relationship between antioxidant behavior and the protein structure, which produce a partial increase but a remarkable total decrease of protein antioxidant capacity.

The reciprocal relationship between molecular stability and flexibility, two elements of the dynamic of molecular changes, used to explain loss of biological activity for proteins [25], [26], could help us to understand the induction of an incipient antioxidant response and the important reduction in the RS component observed here with albumin modified with acrolein and glucose. If the changes introduced in albumin increase the protein stability (rigidity of the molecule), a reduction of flexibility and then a corresponding reduction in the RS component can be expected, as was observed here (Figure 9). The loss of flexibility apparently does not exclude the initial increased AC produced by the structural modifiers, possibly because it was derived from some intact Cys 34 residues.

**Figure 10:**
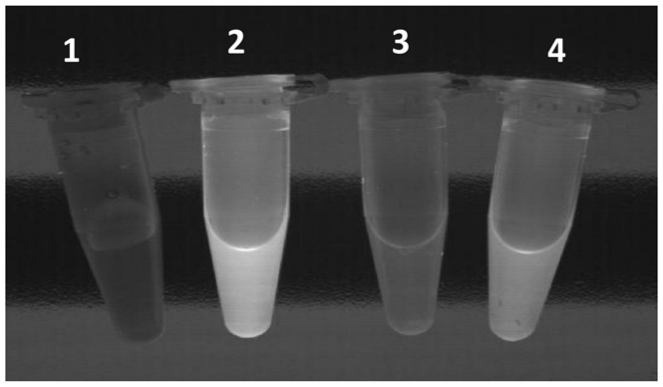
Fluorescence enhancement of 8-Anilino-1-naphthalene sulfonate (ANS) in the interaction with albumin. Different tube contents are: 1. Aqueous solution of ANS without protein; 2. ANS upon binding to normal albumin; 3. ANS upon binding to acrolein-treated albumin; 4. ANS upon binding to glycosilated albumin. When irradiated with UV light, intense fluorescence can be observed in tube containing ANS-normal albumin and a diminished fluorescence from the tubes with ANS-modified albumin; meanwhile no glow is observed in the tube with an aqueous solution of free ANS. The picture was taken directly on the UV transilluminator as described in Material and Method section.

**Figure 11:**
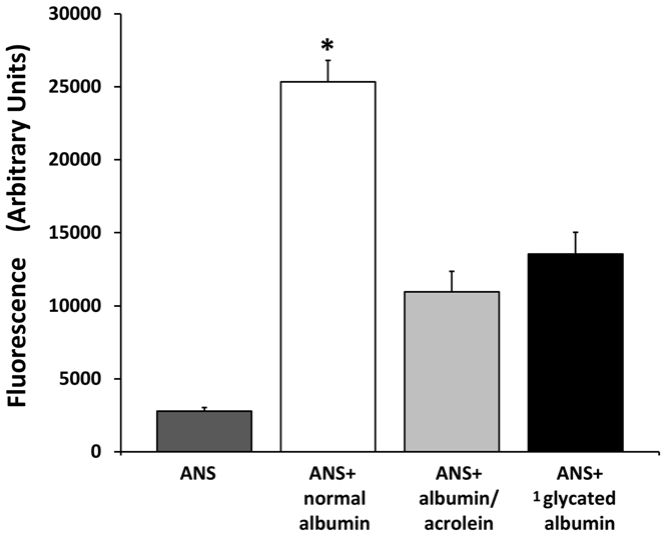
Measurement of the fluorescence of 8-Anilino-1-naphthalene sulfonate (ANS) binding to modified and not modified albumin. Normal albumin presents a high level of fluorescence. Albumin modified with acrolein and partially glycated albumin quenches the level of fluorescence by more than two times and nearly two times respectively. Results are expressed as mean ± SD (n = 8). *P<0.001 vs. all the groups. ^1^Partially glycated as described in the Material and Methods section.

With the objective of revealing changes produced in the native structure of albumin by the modifications described previously, binding studies were performed with the hydrophobic compound 8-Anilino-1-naphthalene sulfonate (ANS), a sensitive probe for partially folded intermediates in protein-folding pathways. Results reveal how the fluorescence of ANS increases substantially when it is bound to normal albumin, (providing evidence of a partially folded state in the commercial albumin). In contrast, the same albumin modified with acrolein and glycosilated albumin both reduced substantially the fluorescence of ANS excited by UV light. This was probably due to structural changes and the reduction of hydrophobic core regions that are inaccessible to the dye (Figure 10). Independent experiments confirm quantitative reductions in the ANS fluorescence (Ex: 355 Em: 460) of modified albumin (Figure 11). A reduction in hydrophobicity of albumin showed a positive correlation with the AC; however, a drastic reduction in the RS accompanied the same change in hydrophobicity (Figures 8 and Figure 9, respectively). Changes in ANS fluorescence bonding to albumin as a result of previous albumin transient UV light exposure were negligible. Taken together, these data indicate how albumin responds to different stressors with more antioxidant capacity and how pronounced and permanent changes in the structure can negatively modify the antioxidant response (RS).

### An Approach for the Construction of a Model for the AC and RS of Proteins

It has been demonstrated that the free thiol groups in HSA (Cys-34) can interact with reactive oxygen species (ROS) like hydrogen peroxide (H_2_O_2_) and peroxinitrite (ONOO-) to form a sulfenic acid derivative (HSA-SOH) [10]. Sulfenic acid has been described as very probably the most potent of all peroxyradical trapping antioxidants [27]. The sulfenic acid can be oxidized to sulfinic acid (RSO_2_H) and sulfonic acid (RSO_3_H), and can also react with thiols, providing a mechanism for disulfide formation [28]. In addition, sulfenic acid can undergo self-condensation to yield the correspondent diallyl thiosulfinate; these can undergo Cope elimination at room temperature to give 2-propensulfenic acid and thioacrolein [27]. From the diversity of possible reactions, some of these make a hypothetical cycle possible.

Using some previously described information and evidence from the present work it is possible to construct a theoretical model to explain the possible mechanism of the antioxidant capacity and RS of proteins. We present a specific alternative, and try to simplify and summarize the available information.

**Figure 12:**
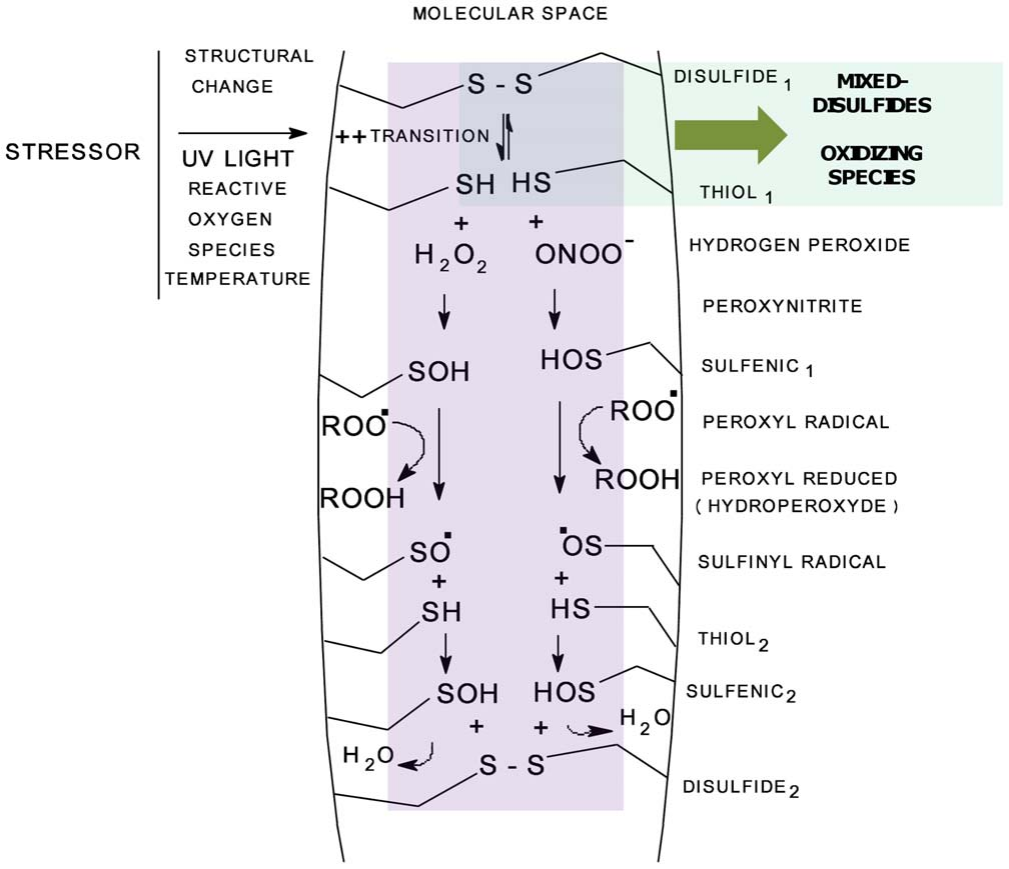
Theoretical model to explain the effect of a stressor on the changes in AC and RS of Human Serum Albumin (HSA). In some places on the molecular space, stressors induce a transition state between disulfide (DISULFIDE_1_) and thiol groups (THIOL_1_). Reactive oxygen species (ROS) such as hydrogen peroxide (H_2_O_2_) and peroxynitrite (ONOO-) can react with thiols to form a sulfenic acid derivative (HSA-SOH) (SULFENIC_1_). The Sulfenic group is an efficient reducer of peroxy radicals (ROO) and reacts with it to form a sulfinyl radical derivative (HSA-SO) and the correspondent hydroperoxyde (ROOH). A new sulfenic acid derivative (SULFENIC_2_), this time formed by the reaction of previously formed HSA-SO^.^ with new thiol groups (THIOL_2_), completes the cycle with the formation of additional disulfides (DISULFIDE_2_). Protein requires space to move itself between transition states (flexibility). The precise localization of cysteine residues facilitates the cycle. The driven force to move the entire system depicted should be the more favorable thermodynamic molecular state derived from the primary structure. Pale green  =  passive component; pale violet  =  active component or structural change dependent. Intersection is common to both components.

In accordance with this model (Figure 12), a stressor introduces instability into the core of a protein that promotes the transition (structural perturbation) between disulfide and SH groups. In the present work, thermal treatment and UV light as stressors and DTT all increased the antioxidant capacity of albumin by a high magnitude (Figure 1, Figure 2 and Figure 5), whereas in addition ribonuclease exposure to UV light clearly liberates SHs groups that then were detected (Figure 6). Thiols will then interact with reactive oxygen species (ROS), re-oxidize or form mixed disulfides. Although it is a critical point in the mechanism and represents a primer, it is strictly part of the passive antioxidant capacity of a protein. In contrast, in a second dynamic phase, a sulfenic acid derivative formed from the reaction between ROS and thiols can reduce free radicals such as peroxyl radicals (ROO) with high efficiency, as has been demonstrated [27], generating sulfinyl radical derivatives (RSO) as the product of the reaction. Then, a second stock of sulfenic acid can be recycled from RSO^.^ if enough SHs are available, which will be converted to a new S-S group (Figure 12). It is possible to assume that flexibility is necessary, allowing the proposed instability induced by the stressor. In addition, data from experiments with ANS it is possible to suggest that hydrophobic molecular regions should be implicated in the antioxidant response of proteins, although quantitative differences should exist between native and non-native albumins.

If the native configuration represents the more favorable energy state for a protein in accordance with Anfinsen's thermodynamic hypothesis [18],[19], then it is possible to hypothesize that the thermodynamically most stable native state must represent the more favorable antioxidant state and the driving force that moves the protein thiol groups towards the most favorable location. It is very important to explain the tight interdependence between structure and antioxidant function, but in addition it could help us to understand why a protein assumes its particular configuration.

### Implications

No matter what the precise mechanism of protein antioxidant capacity is, including the RS component, the existence of this extraordinary behavior has several implications by itself. The entire antioxidant capacity of a system such as serum, plasma or any tissue may be conveniently evaluated only if the entire antioxidant capacity of proteins is taken into account. The antioxidant capacity of any biological component is apparently much more complex than was previously supposed. A biological sample should present some of the several active elements with antioxidant capacity at the same time: antioxidant enzymes (SOD, glutathione peroxidase, etc.); primary antioxidants such as ceruloplasmin and ferritin; the soluble non-protein-dependent antioxidants (vitamin C, glutathione, etc.); a protein antioxidant capacity dependent on SH and other residues (passive component); and the Response Surplus (RS) described here (active component).

The importance of recognizing all the components of the antioxidant capacity becomes critical when the antioxidant status of a biological sample from patients suffering an illness is evaluated. A large number of studies support the hypothesis that oxidative damage to DNA, lipids and proteins may contribute to the development of cardiovascular disease, cancer, and other degenerative disorders such as arteriosclerosis, diabetes, Alzheimer's and also in ischemia/reperfusion injury, ethanol intoxication, liver steatosis and ageing. If a new component of the biological antioxidant capacity not previously considered is finally recognized, it will likely radically change the knowledge obtained from this theme. For example: the destabilization of a native protein in a critical site in the body should result in the drastic reduction of its antioxidant potentialities and should become the preliminary step before aggregation.

Additionally, RS should help us understand inconsistencies in previous reports when human plasma total antioxidant activity has been evaluated. Although some reports have emphasized the loss of albumin antioxidant activity as the result of structural changes [17], [29], it seems clear that the evaluated component in these works correspond to the relationship between mercaptalbumin (reduced form) and non mercaptalbumin (oxidized) alternative forms of HSA. From the present work, we can deduce that the evaluation referred to corresponds to the passive antioxidant component, attributable only to the oxidation of SH groups and in particular Cys 34. When in the work of Kawakami et. al. the hydroxyl radical scavenging activity of reduced and oxidized HSA was studied using ESR spectroscopy (ESR), reduced albumin [HSA (red) % = 73%] quenched up to 68.7% of the ESR hydroxyl radical signal, while the oxidized form [HSA (red) % = 8%] reduced the signal by 54.4% [17], and the authors concluded that oxidation of HSA diminished its radical scavenging activity. We suggest that the scavenging properties of HSA described are only partially interpreted. If the reduced albumin quenched 68.7%, then oxidized albumin must quench only 8% of the ESR signal and not 54.4% if the total antioxidant potential of the protein had been dependent only on the oxidation of SHs groups. We can see in these results indirect evidence of the existence of the antioxidant component RS. Oxidized albumin reduced the ESR signal more than predicted by the SHs proportion because an unpredictable component was present. An additional component such as RS is necessary to explain how a reduction of 65% in SHs groups of HSA can reduce less than 15% of its radical scavenging activity.

Finally, we found that some texts include reports of “unidentified antioxidant(s)” that represent a percentage of the total antioxidant capacity of the plasma [30], [31], [32], [33]. From the data presented in this work it is possible to suggest that the value of unidentified and not yet clearly defined antioxidant reported previously corresponds to the indirect influence of components of the protein antioxidant capacity and in particular RS. This is extremely important because even though a soluble non-protein component of a biological sample could be evaluated alone, the influence of a protein antioxidant reserve as a part of a whole should be decisive and must be taken into account.

## Materials and Methods

All compounds used were from Sigma Chemical Company (St. Louis, MO, USA) and of the highest purity available. 8- Anilino-1-naphtalenesulfonic acid ammonium salt 95%; Horseradish peroxidase Type I (E.C. 1.11.1.1.7); carbonic anhydrase E.C. 4.2.1.1 (Isoform CA-I, 29 KD from bovine erythrocytes); hydrogen peroxide 30% (w/w); sodium hypochlorite solution 10–13%; acrolein (2-propenal) 99%; HEPES (N-(2-Hydroxyethyl)piperazine-N'- (2-ethanesulfonic acid) 99.5%; p-iodophenol; luminol (5-Amino-2,3-dihydro-1,4-phtalazinedione); albumin from human serum (Lyophilized powder, 99%, essential fatty acid free, prepared from essentially globulin free albumin; albumin from Bovine Serum (fraction V Essentially fatty acid free and globulin free); bovine pancreatic ribonuclease (RNase) E.C. 3.1.27.5.; proteinase K (from *Tritirachium album*) E.C. 3.4.21.64; human insulin (recombinant 5.8 KD). Trolox (6-hydroxy-2, 5, 7, 8-tetramethylchroman-2- carboxylic acid) was obtained from Sigma-Aldrich Inc., USA.

Ultrafree-MC (Amicon, Millipore Corporation, Bedford, MA 01730, USA) Centrifugal Filter device, 5000 and 10000 NMWL cut off.

### Antioxidant Capacity System

In the present work the antioxidant capacity (AC) was measured using an enhanced chemiluminescence based assay. The reaction mechanism (Hydrogen Atom Transfer or HAT) allowed measurement of the potential of an antioxidant to quench free radicals by hydrogen donation. In real time it is possible visualize the individual contribution of each component to the total AC. Horseradish peroxidase (HRP) is an enzyme that catalyses the decomposition of peroxides and forms free radicals. Peroxidase/H_2_O_2_ mixtures have been used to generate free radicals [34] from several substrates. Hydrogen peroxide removes two electrons of the enzyme, and each of these is replaced in two one-electron steps, in each of which a substrate molecule forms a radical [35]. Oxygen consumption for the sodium horseradish peroxidase/hydrogen peroxide system and free radicals generation both have been documented [36]. Radicals formed, peroxides and oxygen oxidize luminol, producing light that can be detected and measured. Given that a constant rate of radical generation can be achieved, the light emission depends on constant production of free radicals. Enhanced chemiluminiscence has been used to measure the antioxidant capacity in biological fluids [37]. The system used in the present work contains Horseradish peroxidase (5 µU/l), hydrogen peroxide 30% w/w (3.0 µM), substrate p-iodophenol (25 µM) and luminol (300 µM from a stock solution of 10 mM in DMSO). A reaction mixture containing perborate base buffer prepared with sodium tetraborate (100 mM) and sodium carbonate (100 mM) was used. Intra-assay variation for fifteen samples analyzed in triplicate was 0.29–5.2%, with a mean of 4.05%. The luminometer display and complementary information are depicted in supporting information Figure S2.

### Antioxidant Standard Preparation

Using the procedure described, a standard curve was achieved using increasing amounts of the water soluble tocopherol analogue Trolox (6- hydroxy-2, 5, 7, 8-tetramethylchroman 2-carboxylic acid). Standards of Trolox were prepared in milli-Q water (Millipore Corporation, Bedford, MA 01730, USA) before initiating each experiment and starting with a stock solution of 80 µM. The antioxidant capacity of test solutions through the experiments in the present work is expressed as nM of Trolox (Trolox Equivalent Units or TEU nM). An approximate amount of 320 nM of Trolox is enough to suppress completely the chemiluminiscent signal and correspond to the maximal antioxidant capacity (C) achieved. One standard curve was prepared before each experiment.

### Protein Samples Conditioning

After the treatment, all protein samples were processed to remove the low molecular components of the reaction mixture. Before the incubation period, the protein samples were placed in microcentrifuge filters (10 KDa cutoff; 5 KDa cutoff for insulin) (Microcon, Millipore, Bedford, MA, USA), and centrifuged at 5000 g for 1 hour. The filter residues were washed and dialyzed two more times and dissolved in phosphate buffer pH 7.4. With the objective of removing possible components adsorbed to the proteins, samples were precipitated with 1.5 ml of chloroform and methanol (2∶1). After agitating thoroughly, samples were centrifuged at 3000 g for 10 minutes and the organic phase discarded. The protein residue was dried (nitrogen) and dissolved in PBS pH 7.4.

Finally, the samples were centrifuged at 5000 g for an additional 10 minutes and soluble residues separated; the protein concentration was determined using the method of Bradford [38]. Aliquots containing 60–500 µg of protein were added to the reaction mixture in the luminometer cuvette after the signal was stable.

The residues of solvent remaining after dialyze samples were collected and placed to react with Folin-Ciocalteu reagent to monitor protein fragmentation. In addition to amino acid residue observation, possible light scattering due to aggregation was measured at 400 nm in a spectrophotometer with the objective of taking control of possible aggregation of irradiated proteins.

### Structural Stressors

#### Oxidative stress

To probe the increase in antioxidant capacity of a protein subject to a previous oxidative challenge, albumin samples were first incubated at 37°C in 50 mM phosphate-buffer saline (PBS), pH 7.4 with either 2 mM of hydrogen peroxide (H_2_O_2_) or 500 µM sodium hypochlorite (NaOCl) for 10 minutes, or exposed to UV light (254 nm). The final volume in all the experiments was 1 ml and the protein concentration was 1 mg/ml. In the experiments where the antioxidant capacities of the proteins are compared and for correlation with the half life, 10 µg/ml of protein were used for each assay (Carbonic anhydrase, albumin and insulin). RNase (Bovine pancreatic ribonuclease) was used at a concentration of 1 mM, and the treatment with UV light was as described for albumin.

#### Temperature as structural stressor

To demonstrate the influence of temperature on the AC and RS of HSA, samples subject to several temperatures ranging from 10 to 50°C±0.1°C were tested, using a Peltier System. Albumin stock solutions with a concentration of 100 mg/ml were placed with the respective controls for each temperature point from 10 to 50°C. At this concentration aggregation appear close to 50°C. Aliquots from stock solutions were taken for carried out experiments. The free radical production was monitored and results were constant with a variability of less than 2%. The thermally treated samples were placed at defined temperatures in the presence of the reaction mix, and the antioxidant capacity was measured using enhanced chemiluminescence, as mentioned previously (Antioxidant capacity system).

#### Treatment with UV light

UV light was used in the present work as a particularly clean stressor, although it does not minimize the possibility of direct structural effects on the protein derived from the oxidative stress. Oxidation of proteins in aqueous solutions with UV light leads to generation of several reactive oxygen species [39], [40]. Indeed, we thus emphasize the close relationship between structure and protein antioxidant capacity. In addition, we verify the results and in particular the phenomenon of Response Surplus (RS) with the use of ROS such as structural modifiers. The UV light exposed samples were placed at a distance of 5 cm from a source lamp in a 3 ml capacity 1 cm wide quartz cuvette at variable periods of time for each experiment. The energy of the light used in the experiments was calculated at 1 µW/cm^2^. A Peltier system was used to maintain the temperature at 25±2°C. The use of selected long wave and optimal conditions of protein response were determined experimentally.

#### Treatment with Proteinase K (PK)

In the experiments exploring the influence of proteolysis on the antioxidant capacity of HSA, samples containing 1 mg/ml of protein were incubated at 37°C with PK (300 mU), 50 pmol Tris buffer pH 7.4 for 15 and 60 minutes. The remaining protein was precipitated by the addition of 2 ml ethanol and 0.12 ml of 4.75 M acetate buffer pH 5.0, and analyzed by the Biuret reaction. More than 90% of hydrolysis was achieved with 60 minutes of incubation time. At the end of the incubation period, samples were subjected to the procedure described previously in the “Protein sample conditioning” section.

#### Treatment with dithiotrithol (DTT)

To determine the antioxidant capacity of HSA reduced with DTT, samples containing 1 mg/ml of protein were incubated with DTT (10 mM) until complete reduction was achieved, and then filtered as described in the “Protein samples conditioning” section. Antioxidant capacity was achieved immediately. Reduction was followed by the release of 5- mercapto-2-nitrobenzoic acid (MNB); more details are in the Ribonuclease thiol group section. No additional albumin reduction was detected after 1 minute of incubation, but additional incubation time produced protein fragmentation and hence loss of protein during the washing procedure.

#### Treatment with acrolein

The irreversible modification of albumin with acrolein was assayed as described previously [26] for insulin. As previously described, HSA was prepared containing 1 mg of protein in 1 ml of Milli Q water adjusted to pH 8.0 with 0.5 M Tris base and incubated for 4 h at 7°C with 300 µM acrolein (Sigma. St. Louis, MO, U.S.A). At the end of the incubation, the pH of all of the samples was adjusted to 7.4, placed in microcentrifuge filters (10 KDa cutoff) (Microcon, Millipore, Bedford, MA, USA), and centrifuged at 5000 g for 45 min. The filter residues were washed as described previously in the “Protein sample conditioning” section and refrigerated until the measurement procedure was carried out.

#### In vitro glycation of HAS

Glycated albumin was prepared as described previously [16] with modifications. Briefly, HSA was dissolved in PBS, pH 7.4 to yield a stock solution of 40 mg/ml. Dilutions with glucose prepared in PBS results in an incubation mixture containing 10 mg/ml HSA with 100 mM glucose. After filtration (0.22 µm filters, Millipore), the mixture was incubated for 1 month at 37°C. Unbound glucose was removed by extensive dialysis against PBS, pH 7.4, and stored in the dark.

### Ribonuclease Free Thiol Groups Produced by UV Exposition

For evaluation of the SHs punctual contribution to the antioxidant capacity of a protein, the free thiol groups of ribonuclease resulting from the UV treatment were measured using Ellman's reagent. Briefly, 5,5′-Dithiobis(2-nitrobenzoic acid) 2.5 mM in 0.2 M phosphate buffer pH 8.0 was mixed with 50 µl of sample (ribonuclease 1 mM) and 750 µl of 50 mM phosphate buffer pH 8.0. Freshly prepared Ellman's reagent (250 µl) was added and the reaction proceeded for 15 minutes at room temperature in the dark. The release of 5-mercapto-2-nitrobenzoic acid (MNB) was monitored at 412 nm in a Beckman DU-600 spectrophotometer. MNB was quantified using an extinction coefficient of 14150 mol-1 cm-1 [41]. The results were expressed in µmol/mg protein.

### 8-Anilino-1-Naphthalene Sulfonate (ANS) Binding

The binding of the hydrophobic probe ANS to protein was verified to assess small changes produced in the native structure of albumin [42]. Albumin samples prepared with acrolein and glucose as described previously were suspended in buffer at a concentration of 0.5 mg/ml in 50 mM HEPES, 100 mM KCL, pH 7.8. ANS was prepared just before assays and was used at a final concentration of 0.1 nM. The image of aqueous solutions of ANS excited by ultraviolet light was achieved using a BioDoc-It System with UV transilluminator (UVP, Inc., Upland, California, U.S.A.). Fluorescence measurements were performed on a BioTek plate reader (BioTek Instruments, Inc.). The excitation wavelength was set at 355 nm and the emission taken in 460 nm. The temperature was fixed at 30°C.

### Statistical Analysis

In the present work, groups were compared using the one-way ANOVA test followed by Dunet's test. Results were considered to be statistically different when p<0.01.

## Supporting Information

Figure S1 Antioxidant capacity and protein half life. Three proteins tested (human insulin, bovine albumin and carbonyc anhydrase) presented an inverse relationship between antioxidant capacity and the protein′s half life. Antioxidant Capacity as Trolox nM concentration used as standard (Trolox Equivalent Units.
(0.68 MB TIF)

Figure S2 Assay procedure complemented information. The assay procedure is described as follows: to the luminometer cuvette, 800 µl of phosphate buffer solution pH 7.4 and 200 µl of reaction mix are added (dilution 1∶10 with PBS). Before the cuvette is placed in the luminometer (BioOrbit, Turku, Finland), 100 µl of Horseradish peroxidase (5 µU/l) are added and then mixed well, allowing the light emission to stabilize. The temperature of the system was maintained at 25±2°C. The chemiluminiscent signal remains stable for several minutes with an output of around 1600 mV. With the stable maximum signal the instruments carried out a run of precisely 3 minutes. The correspondent area below the curve represents the no-add antioxidant value (N). Any reductions of the absolute area below the curve during an identical run using standard or sample correspond to the antioxidant capacity of the test solution and (C) represents the remaining area.
(0.15 MB TIF)

Table S1 Antioxidant capacity and protein cysteine content. A correlation between antioxidant capacity and the total amount of cysteine residues per gram of protein (calculated) could be observed for human insulin, bovine albumin and carbonic anhydrase.
(0.24 MB TIF)
