# Supplementary figures and images for: Protein Antioxidant Response to the Stress and the Relationship between Molecular Structure and Antioxidant Function
Source: PLoS One. 2010 Jan 29;5(1):e8971. doi: 10.1371/journal.pone.0008971 (PMC2813298; doi:10.1371/journal.pone.0008971)

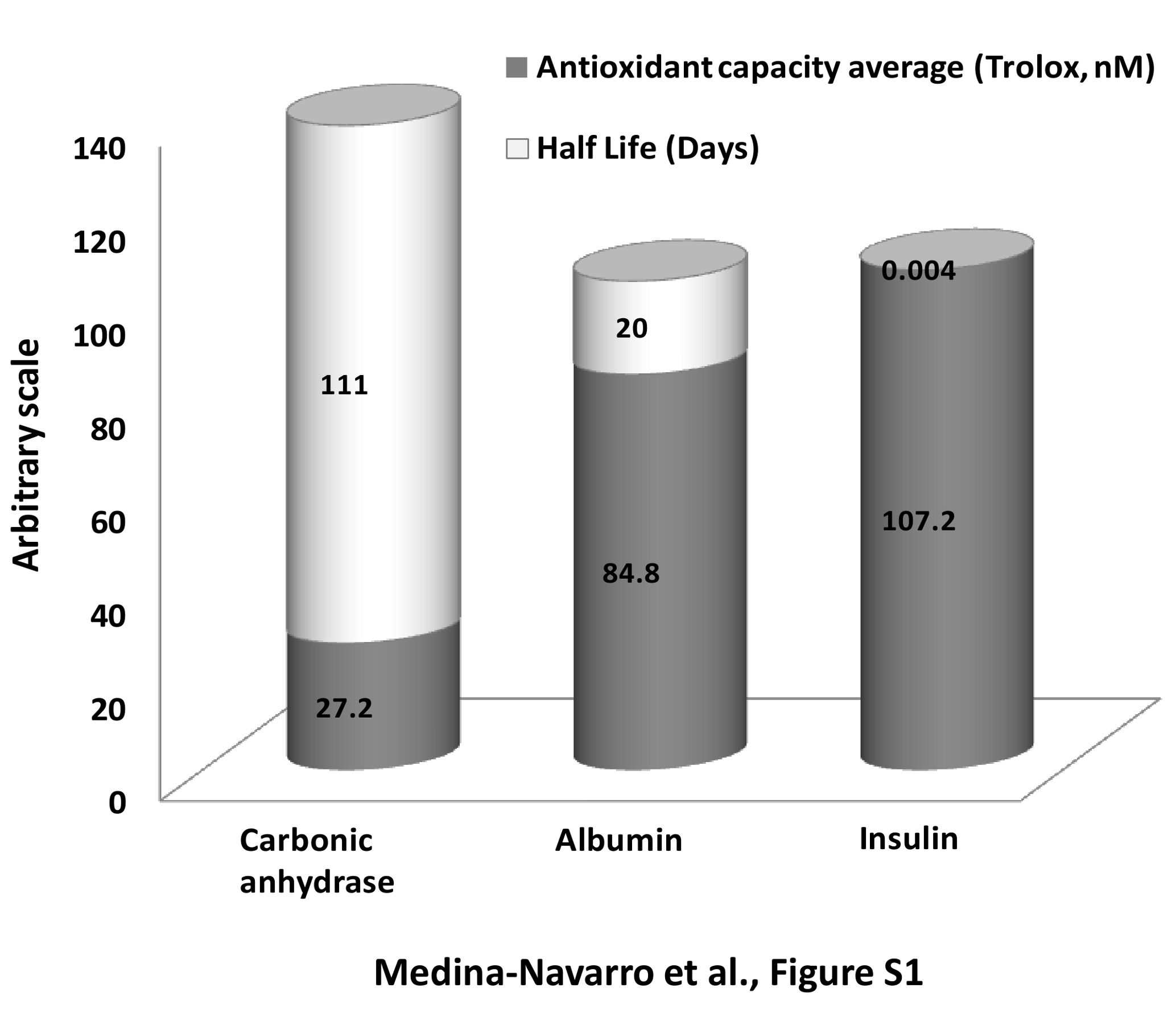

Supplement: Figure S1 — Antioxidant capacity and protein half life. Three proteins tested (human insulin, bovine albumin and carbonyc anhydrase) presented an inverse relationship between antioxidant capacity and the protein′s half life. Antioxidant Capacity as Trolox nM concentration used as standard (Trolox Equivalent Units. (0.68 MB TIF) [file pone.0008971.s001.tif]

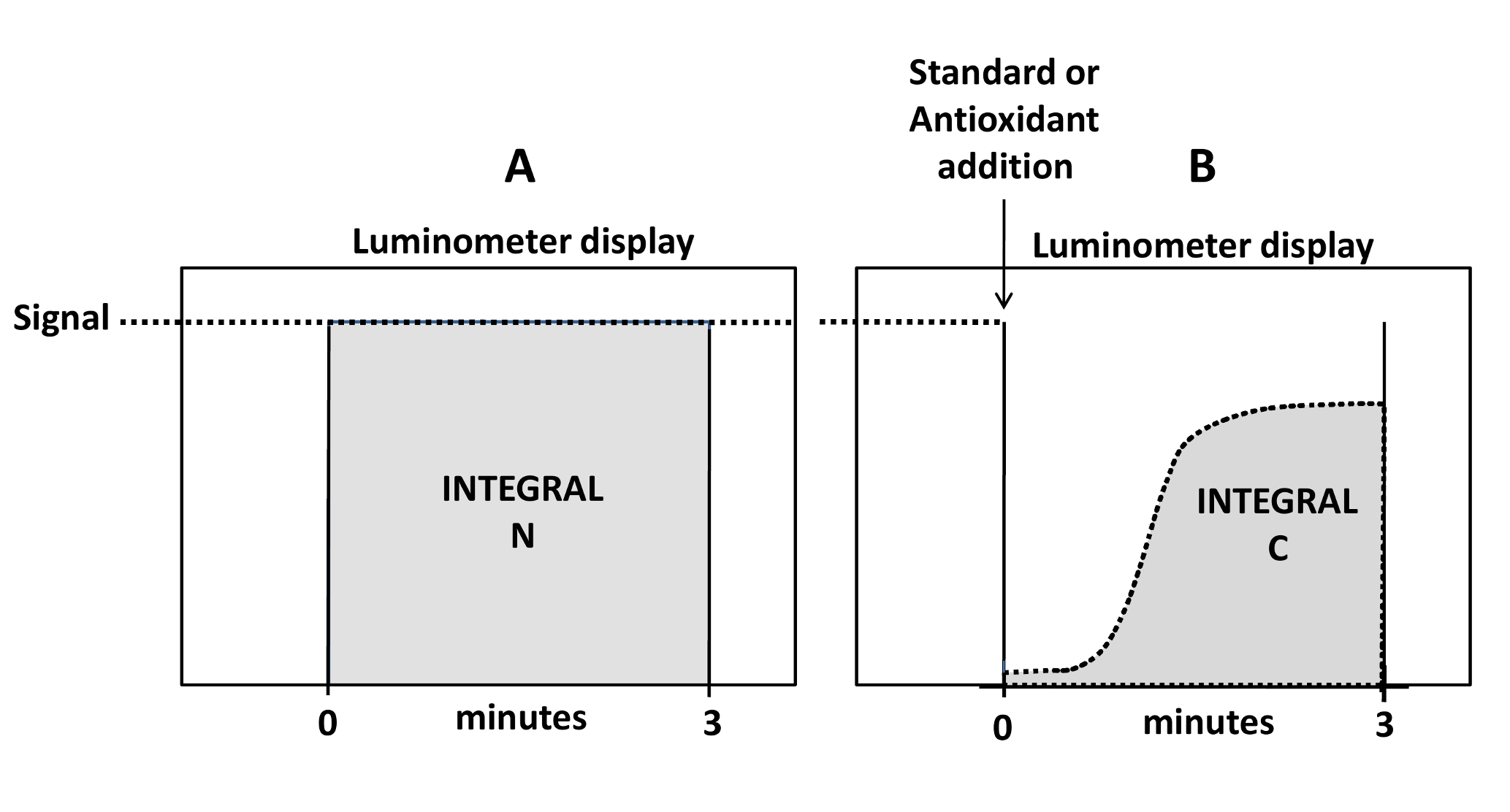

Supplement: Figure S2 — Assay procedure complemented information. The assay procedure is described as follows: to the luminometer cuvette, 800 µl of phosphate buffer solution pH 7.4 and 200 µl of reaction mix are added (dilution 1∶10 with PBS). Before the cuvette is placed in the luminometer (BioOrbit, Turku, Finland), 100 µl of Horseradish peroxidase (5 µU/l) are added and then mixed well, allowing the light emission to stabilize. The temperature of the system was maintained at 25±2°C. The chemiluminiscent signal remains stable for several minutes with an output of around 1600 mV. With the stable maximum signal the instruments carried out a run of precisely 3 minutes. The correspondent area below the curve represents the no-add antioxidant value (N). Any reductions of the absolute area below the curve during an identical run using standard or sample correspond to the antioxidant capacity of the test solution and (C) represents the remaining area. (0.15 MB TIF) [file pone.0008971.s002.tif]

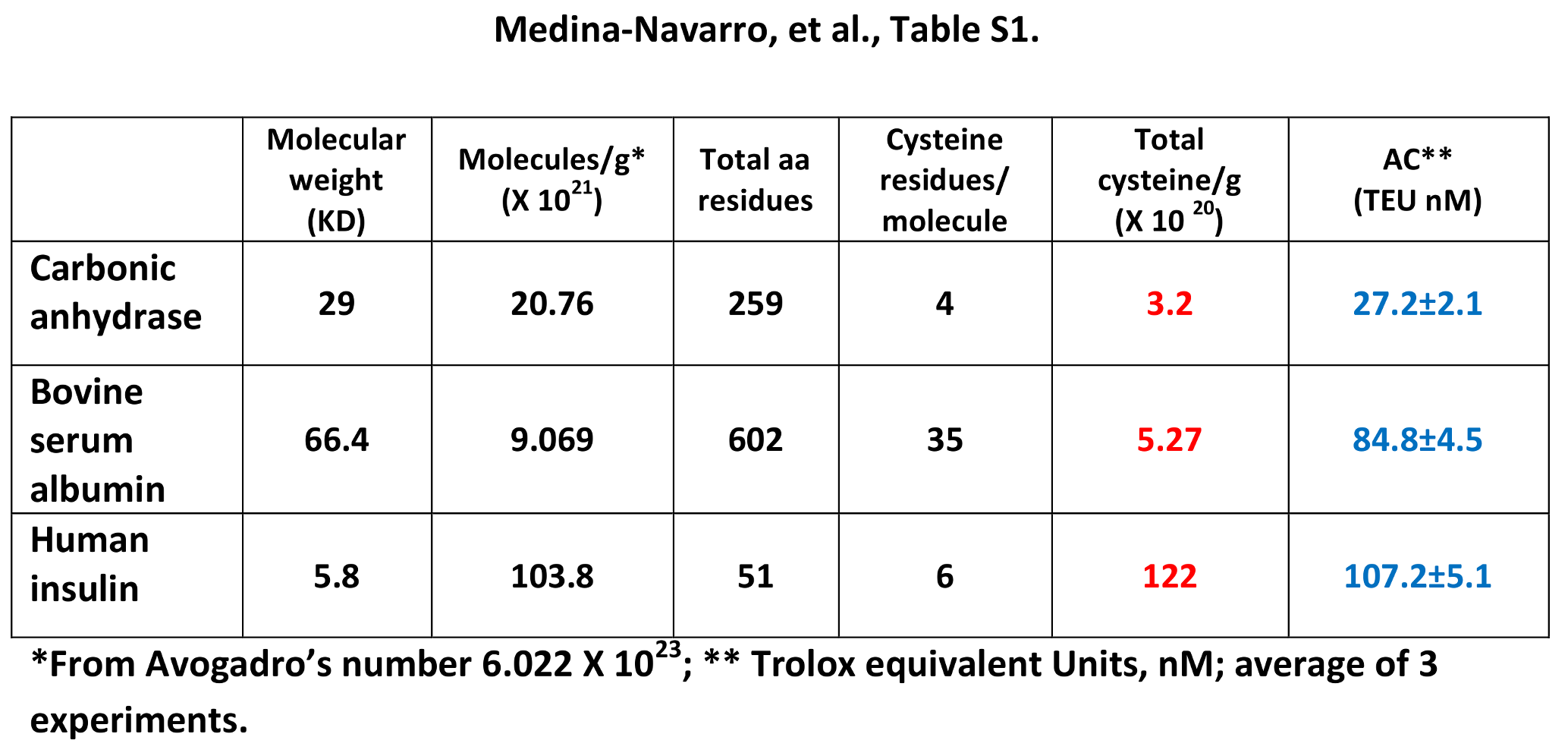

Supplement: Table S1 — Antioxidant capacity and protein cysteine content. A correlation between antioxidant capacity and the total amount of cysteine residues per gram of protein (calculated) could be observed for human insulin, bovine albumin and carbonic anhydrase. (0.24 MB TIF) [file pone.0008971.s003.tif]
